# Nanoparticles in Agroindustry: Applications, Toxicity, Challenges, and Trends

**DOI:** 10.3390/nano10091654

**Published:** 2020-08-23

**Authors:** Luis A. Paramo, Ana A. Feregrino-Pérez, Ramón Guevara, Sandra Mendoza, Karen Esquivel

**Affiliations:** 1 Graduate and Research Division, Engineering Faculty, Universidad Autónoma de Querétaro, Cerro de las campanas, C.P. 76010, Santiago de Querétaro, Qro., Mexico; luissofonsolaps@gmail.com (L.A.P.); feregrino.angge@hotmail.com (A.A.F.-P.); ramonggg66@gmail.com (R.G.); 2 Programa de Posgrado en Alimentos del Centro de la República (PROPAC), Research and Graduate Studies in Food Science, Chemistry Faculty, Universidad Autónoma de Querétaro, Cerro de las Campanas, C.P. 76010, Santiago de Querétaro, Qro., Mexico; smendoza@uaq.mx

**Keywords:** nanotechnology, nanoparticles, agriculture, metabolomics

## Abstract

Nanotechnology is a tool that in the last decade has demonstrated multiple applications in several sectors, including agroindustry. There has been an advance in the development of nanoparticulated systems to be used as fertilizers, pesticides, herbicides, sensors, and quality stimulants, among other applications. The nanoencapsulation process not only protects the active ingredient but also can affect the diffusion, interaction, and activity. It is important to evaluate the negative aspects of the use of nanoparticles (NPs) in agriculture. Given the high impact of the nanoparticulated systems in the agro-industrial field, this review aims to address the effects of various nanomaterials on the morphology, metabolomics, and genetic modification of several crops.

## 1. Introduction

In the agro-industry area, nanotechnology is commonly used for generating products such as fertilizers, herbicides, pesticides, fungicides, and nano-sensors [1]. These advances can help overcome future demands in agriculture, increasing quality and crop yield, reducing pollution caused by chemicals, or even protecting crops against environmental stresses [2].

While there are interesting applications of nanotechnology in agriculture, it is well known that we still do not fully understand the negative effects that these materials can generate in the environment [3], particularly in plant and living organisms. Many types of research have shown that nanoparticles (NPs) at high concentrations can generate toxicological effects on crops such as lettuce, tomato, wheat, and cucumber, just for mention a few of them [4,5,6,7].

Nanotechnology has many benefits that deserve to be explored for the solution of certain problems; however, we must be aware that its application without care can lead to a series of issues to the plants, animals, and finally to humankind. As addressed by Mishra et al. (2019) [8], it is important to increase the nanoparticle safety awareness and build strong regulation systems so that we can apply nanotechnology with safety and avoid environmental disasters. For example, the production of nanoparticles (NPs) has been raised to the industrial level: more than 10,000 Tn of titanium dioxide (TiO_2_) NPs were produced worldwide in 2010 [9], and it was estimated that 500 to 1000 Tn of CeO_2_, FeOx, AlOx, ZnO NPs, and carbon nanotubes were produced every year only in the European region [10].

That is why in this review, we address a brief compendium of recent studies that have been made to try to understand the effects of diverse NPs properties when they interact with several types of plants, leading into some morphology, metabolomics, and genetic modifications.

## 2. Nanomaterials Classification

Nanomaterials are characterized by having a size between 1 and 100 nm in at least one of its dimensions (length, height, and width) [11,12]. They display different properties (optical, electronic, and chemical, among others) from their counterpart in bulk, due to a greater surface area as well as the quantum properties that are presented on this scale [13]. These new properties have allowed us to find unique applications.

Nanomaterials can be classified depending on how many of their dimensions are in the macro scale: 0D includes the NPs whose dimensions are all on the nanoscale, 1D are the nanofibers and nanowires that only have one dimension in the macroscale, 2D covers nanosheets and thin films, and finally, 3D represents the materials in bulk [14].

Apart from classification by dimension, nanomaterials can be classified by their chemical nature. This classification contains 4 main categories of nanomaterials: carbon, ceramic (metal oxides), metal, and polymeric compounds [13]. Carbon-based nanomaterials include structures such as fullerenes, graphene, and carbon nanotubes (CNTs) [15]; ceramic are inorganic solids made of metal–oxide compounds such as TiO_2_, ZnO, and FeO_2_ [16]; and metals include nanomaterials based on Au, Ag, Cu, and Ni. Organic nanomaterials include dendrimers, which are derived from organic NPs that are generally symmetrical to the nucleus [15].

### 2.1. Metal Oxide NPs

Several metal oxide compounds offer photocatalytic characteristics such as, TiO_2_, ZnO, WO_x_, SnO_2_, Fe_2_O_3_, CuO, ZrO_2_, and MoO_3_ [17]. When photocatalytic compounds interact with light whose energy is greater than or equal to the bandgap, they promote an electron to the conduction band [18]. This creates a positive hole in the valence band, allowing the excited electron and the hole to generate hydroxyl radicals and other reactive oxygenated species that are involved in degradation reactions [19].

To enhance the photocatalytic activity and production of reactive oxygen species, the metal oxide NPs surface can be modified by metal ion doping or non-metal insertion [20], offering effects such as increased mobility between electron and hole pairs, the formation of new energy states, and an increase in the absorption of visible light [21]. In addition, non-metallic particles can be used to narrow the bandgap or form intra-bandgap states [22].

The degradation processes of the catalysts mentioned above are mainly carried out in wastewater treatments to remove recalcitrant and persistent pollutants such as dyes and pharmaceutical compounds, and the catalysts even work as disinfection agents [23,24,25,26]. The remaining effluents are used for watering some crop fields without any proper legislation [27,28], and no toxicology studies are well substantiated yet, because the remained NPs in the effluents are considered not dangerous in low concentrations. Nevertheless, a new research area is emerging to contribute some broader knowledge to differentiate the pro and cons of using these materials in agriculture systems [29].

### 2.2. Metallic NPs 

Metallic NPs have found many applications—for example, in medical diagnostic, antibacterial, electrodes, and optics. These NPs are comprised of metals such as Au, Ag, Pt, Zn, and Ni (and alloys between metals are also include in this classification [30]). NPs such as gold that are inert at bulk scale become more chemically active when their size is decreased due to a larger spacing between atomic coordinates, which allows their use in catalytic applications [31] 

### 2.3. Carbon-Based Nanomaterials 

This category includes some famous structures such as nanotubes, nanofibers, fullerenes, and graphene, due to their multiple structures and dimensions; these materials are commonly referred to as nanomaterials [32,33]. Their applications include their use in solar cells, structural materials, electronics, and semiconductors, which is mainly due to their interesting properties such as semiconductor behavior, thermal conductivity, high structural properties, and light absorption properties [30]. Carbon nanotubes are also classified depending on their number of walls, which can be single-wall, double-wall, or multi-wall; the number of walls will modify their structural and conductive characteristics [27,34].

## 3. Nanotoxicology 

Nanotoxicology is a sub-discipline of toxicology [35]; it tries to understand the interaction mechanisms of a nanostructured material with a living organism (plants, animals, or even human beings). Some aspects of classical toxicology do not apply to the concept of NPs; according to classical toxicology, “the dose makes the poison”: it is the concentration that determines whether a material can be dangerous. 

However, the toxicity of NPs does not correlate the dose related to mass, not only does the concentration of NPs determine it poisonous level; the size, number of NPs, surface activity, modification, and aggregation are some of the parameters that relate to the poisonous level of NPs [14].

The increasing demand for products containing NPs in production, waste, and water treatment facilities makes it easier for these compounds to enter the environment by releasing the NPs [36]. In addition, it is well known that different types of NPs are being used to improve agriculture systems; they can offer certain advantages, but there is a lack of knowledge regarding the complete toxicological effects of nanoparticles to other biological systems such as fungus, insects, and animals. 

**Figure 1:**
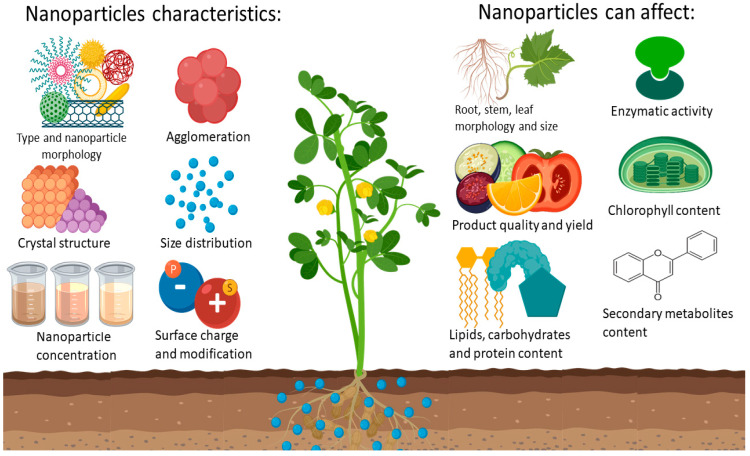
Main characteristics of the nanomaterials and the possible toxicological effects they can induce in crops.

It is difficult to know the effect that certain NPs can cause when they are interacting with a living system. First, the physicochemical characteristics of the NP are the main cause of the generated effects [37]; the morphology, surface charge, concentration, and size distribution are properties that if they are modified individually can cause different results in the same system, as it is shown in Figure 1.

**Figure 2:**
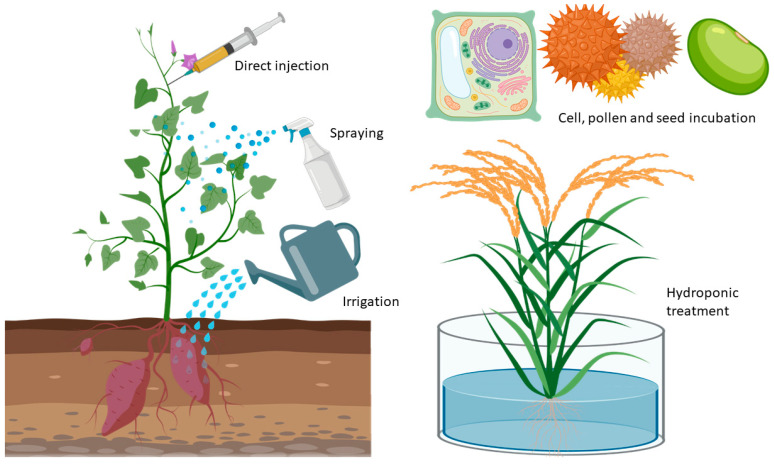
Different types of nanoparticles (NP)/plant exposure methodologies.

The toxicological effects of the NPs are determined not only by the physicochemical characteristics, but also by the experimental design synthesis, the exposure time over the plant, the development phase in which the NP will come into contact with the plant, as well as the means of introduction and interaction of the NPs. There are different methodologies to expose the plant to the NPs (Figure 2), such as the direct injection of NPs into plant tissue [38], NPs spraying into leaves or any other part of the plant [39], contaminating the soil with NPs or irrigating plants with NP suspensions [40], and infecting cellular pollen or seeds [41,42,43].

## 4. Nanotechnology in Agriculture 

**Figure 3:**
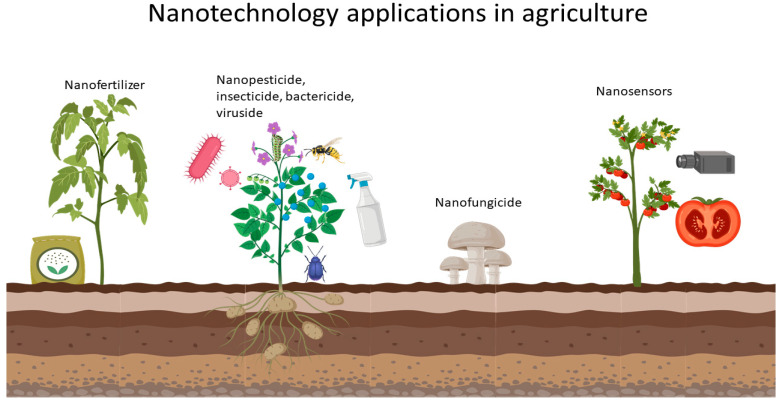
Nanotechnology applications in agriculture.

Nanoparticles find various applications in agriculture; China is one of the most advanced countries in nanotechnology development for agriculture with 28 patents between 2011 and 2015 [44]. Nanotechnology can be applied in agriculture by producing nanofertilizers, nanoherbicides, nanofungicides, and nanosensors (Figure 3). Nanofertilizers promote good development of the crop by helping the necessary absorption of the micronutrients for proper plant development; they can be manufactured of zinc, silica, and titanium dioxide [45], Cu NPs [46], and even polymeric NPs as dendrimers acting as nanocarriers [47]. Nanopesticides generate protection against abiotic-type stresses, their main application falls in the encapsulation of pesticides for controlled release, improving the selectivity and stability of the pesticide, this allows reducing the expenses of pesticides and increasing the lifetime of the active chemical compound [48].

### 4.1. Nanofertilizers

A fertilizer is a natural or artificial substance that contains the chemical elements necessary to improve the growth and productivity of plants and improve natural fertility by overcoming deficiencies of micronutrients [49]. ZnO NPs showed perfect results to overcome zinc deficiency, particularly for rice, by the foliar application of NPs. ZnO also improved the growth and yield parameters and enhanced dehydrogenase enzyme activity [50]. Nanocomposites can also be used for delivering micronutrients such as nitrogen [51]; one example is encapsulated urea modified hydroxyapatite NPs in layers of montmorillonite (clay). These compounds enhanced the yield in rice (*Oryza sativa*) and slow nitrogen release. Bioengineered NPs, such as membrane vesicles, were used to provide Zn to *Brassica oleracea* by foliar fertilization, which resulted in high efficiency and distribution to the protoplast [52].

### 4.2. Nanoherbicides

New nanoformulations intend to reduce the negative impacts that herbicides and insecticides have on the environment; these novel materials try to lengthen and extend the life of these chemicals through controlled release, as well as provide their own protection of the chemical to environmental factors such as degradation by UV radiation [53], as well as a greater selectivity protecting other species of plants, microorganisms, and insects.

Polymeric NPs are the most used for encapsulating chemical compounds; their encapsulation affects the diffusion and release rates of herbicides [54]. Chitosan/tripolyphosphate NPs were used to encapsulate paraquat [55], which is a nonselective herbicide extensively used worldwide. This nanoformulation proved to be less toxic than the pure compound while conserving its effectiveness after encapsulation, showing true potential for protecting other plant species after herbicide treatment.

On the other hand, the use of photocatalytic NPs has been focused on herbicide degradation; ZnO NPs can generate the mineralization of herbicide diquat under sunlight [56], and they can reduce 70% to 90% of glyphosate-based herbicide depending on the herbicide-to-photocatalyst ratio [57]. Titanium dioxide showed a higher degradation rate under solar irradiation of the imazethapyr herbicide, which can be easily decomposed at lower temperatures, showing physisorption on the photocatalyst surface [58]; also, the WO_3_–TiO_2_ nanocomposite showed 100% degradation of the imazapyr herbicide after 120 minutes of UV exposure [59]. 

### 4.3. Nanopesticides

Pesticide toxicity depends on chemical stability, solubility, bioavailability, photodecomposition, and soil absorption [60]; the main objective of nanotechnology is to reduce those effects designing nanocarriers that allow the slow release of pesticides [61].

Nanopesticides based on Cu, (Cu(OH)_2_) have shown negative effects on spinach plants inducing alterations in metabolic profiles, reducing (29–85%) antioxidant molecules such as ascorbic acid, alfa-tocopherol, threonic acid, 4-hydroxybutyric acid, ferulic acid, and total phenolic compounds [62]. These results showed that it is necessary to understand the nanoparticle exposure to living organisms to ensure a safe relationship between nanotechnology and the ecosystem.

Higher yield, durability, and nutrients increase are the advantages offered by certain nanostructured compounds applied to plants. However, the effects of these materials in the long term are yet not fully understand, as these nano-compounds can provide nutrients and protection against pests (insects, plants, bacteria); they also can induce stress in other species of the ecosystem, causing an ecological risk [63].

Similarly to herbicides, polymer nanoformulations are the most attractive as pesticides or ways to encapsulate [60]; again, photocatalytic materials find applications in the degradation of pesticides highly harmful to the environment [64]. Pesticides such as chlorpyrifos, cypermethrin, and chlorothalonil were degraded by TiO_2_ NPs under UVA irradiation, showing complete degradation after 30 min [65]. Meanwhile, Cu-doped ZnO was studied for monocrotophos pesticide degradation; Cu generates an intermediate band to excite an electron from the valence band to the conduction band, increasing optical absorption, decreasing the bandgap, and showing an intense degradation of monocrotophos pesticides [66]. Table 1 shows multiple investigations of the use of NPs in agriculture. 

## 5. Plant Stress

**Figure 4:**
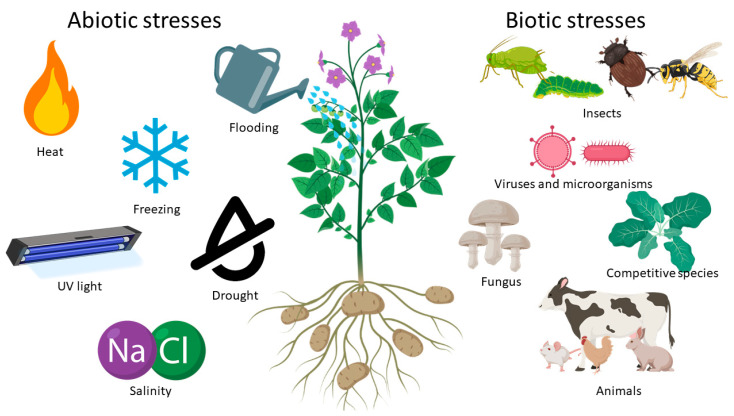
Different types of biotic and abiotic stresses that can affect plants.

Plant stress is defined as any unfavorable condition or substance that affects the metabolism, growth, or development of a plant. A plant organism can enter into a state of stress due to multiple factors; these are divided into two types of categories, abiotic and biotic stresses, as seen in Figure 4 [101]. Abiotic stresses are generated by environmental factors such as light (visible, UV, IR), drought, salinity, temperature, and pH [102].

In the case of biotic stresses, these are produced by living beings, such as microorganisms, insects, viruses, and even other plant species that can induce plant stress. Both types of stresses (abiotic and biotic) can cause damage to the plant [103]. Plants need to adapt to these environmental conditions in order to improve their survival rate by evolution. Plants generate specialized compounds by modifying primary metabolic routes; with these new types of compounds, vegetative organisms can adapt more easily to new conditions. In the worst cases, the organisms cannot overcome the stress, and the survival rates drops drastically [104].

### 5.1. Abiotic Stress

Major yield deficiencies, crop damage, and changes in the growth rate in plants are caused by abiotic stresses such as drought, salinity, the presence of heavy metals, and extreme heat and cold weather; each problem becomes more difficult to overcome due to climate changes and environmental pollution. The effect towards the plant may vary due to the time of exposure, phases of plant growth, and combination with other types of stress such as biotic stresses [105]. These types of environmental stresses are inevitable processes that can result in significant alterations in plant metabolism [106].

#### 5.1.1. Metal Stress

Metals above 5 g/m^−3^ are considered heavy metals; 17 of 53 heavy metals are important for living organisms, such as iron, zinc, manganese, copper, and molybdenum [107]. The rest of the heavy metals are toxic for the environment such as lead, mercury, arsenic, cadmium, and chromium; these metals can affect the biochemical parameters in plants [108]. The presence of heavy metals in plants is a high priority concern to assure secure products for human consumption.

Metals that are classified as micronutrients for plants can also generate toxic effects toward plants when they are present at high concentrations, such as zinc, which inhibits Fe, Cu, Mn, Ca, and Mg uptake in *Oryza sativa (*L.*)* when present at concentrations of 1.5–8.5 mM [109]. Chickpea treated with molybdenum solutions from low to high concentrations (1 × 10^−5^−2 mg∙L^−1^) caused reduced growth at the highest concentration, generated an iron deficiency of young leaves, and reduced the number of flowers, leaves, and pods [110]. Manganese at concentrations of 3 and 6 mM caused growth inhibition for rice seedlings, also increasing the superoxide anion (O_2_^−^)-inducing oxidative stress and causing antioxidant levels imbalance [111].

Pollution caused by heavy metals can be observed around the globe, increasing the negative effects on development, metabolomics, product yield, and quality in plants [112]. The plants can fight heavy metal poisoning by minimizing their uptake, but if the plant cannot deal with the presence of the heavy metals, effects on metabolism transport processes, membranes, and cellular structure can be observed [113].

Reactive oxidant species are produced normally in the plant by the chloroplast, mitochondria, and peroxisomes metabolism, but when facing certain kinds of stress as it has been discussed, the plant can enter in oxidative stress, and heavy metals can also generate this effect in plants [114]. 

Cadmium (Cd), cobalt (Co), and lead (Pb) were tested on pea seeds, inducing a complete failure of germination and seedling growth at high metal concentration [115]. Heavy metal ions have the ability to replace cations in binding sites, inactivating enzymes and producing reactive species, leading to DNA damage, protein degradation, and amino acid oxidation. Antioxidants such as phenolic compounds, ascorbic acid, tocopherol, glutathione, and carotenoids are well known to bind heavy metal ions and reduce and even inhibit reactive species [116]. 

LmSAP is a gene and member of the stress-associated proteins (SAP) that is present in the transgenic tomato, and it enhanced the plant tolerance to heavy metals such as cadmium and manganese. It accumulated more cadmium, copper, and manganese compared to the neutral plant but shows decreased levels of hydrogen peroxide and higher superoxidase dismutase, catalase, and peroxidase activity [117]. 

In this way, it is possible to relate the metals stress to the use of the metals themselves as NPs or even in its oxidized form. 

#### 5.1.2. Nanoparticle Stress

##### Metallic NPs

It is known that reactive oxygen species (ROS) production by metallic NPs depends on structural properties such as the size, shape, and surface area [118]; one example is *Allium cepa* cells, which showed a dose and size-dependent generation of reactive oxygen species, causing enhanced lipid peroxidation and chromosome aberrations when root hair interacts with gold NPs at different sizes (15, 30, and 40 nm) [119].

Ag NPs can release Ag^+^ ions having separately repercussion over vegetal systems; there is no agreement that Ag NPs toxicity is due to the release of these ions or the nanoparticle interaction itself. To address these uncertainties, both NPs and Ag ions toxicity were compared in *Arabidopsis thaliana,* and the results showed that NPs reduced plant rood elongation and vegetative growth while Ag^+^ showed less affectation, showing also a weaker effect on reducing photosynthetic pigment content than the NPs [120]. Arabidopsis thaliana also treated with Ag NPs showed modifications in plasma membrane conductance, also inhibiting root elongation and leaf expansion [121].

Fewer studies for metallic copper NPs (Cu) are made compared to gold and silver NPs, but the interaction on Cu NPs was studied on mung bean (*Phaseolus radiates*) and wheat (*Triticum aestivum*), where it was also found that toxicity is mainly due to NPs rather than the release of cupric ions; Cu NPs were able to reduce the growth rate for both species [122].

Barley plants exposed to Ag NPs enhanced root and shoot length at 0.1 mM, which is attributed to the augmentation of enzyme activities, while higher concentrations (0.5, 1 mM) cause reduced root and shoot lengths. NPs also caused a decrease in chlorophyll content and other photosynthetic pigments [123]; Table 2 shows more studies of metallic NPs interacting with plants.

##### Metal Oxide NPs

Despite not being fully recognized as a type of stress, NPs may fall into the category of abiotic stressors of non-biological origin, since they are not commonly produced by organisms such as bacteria or insects, as mentioned above. Several effects are observed in their interaction with plants; the stress levels and their effects on the plants will be influenced by their characteristics [134].

The uptake and translocation of NPs are many caused by root and leave exposure, where NPs interacting with roots can accumulate on the root surface or interact with root tissue and be translocated by the symplastic pathway, which involves a cell-to-cell transport where NPs with sizes lower than 50 nm are able to get through the cell wall. Transport on this pathway can diffuse passively or actively by transporters in the root plasma membrane [135,136]; alternatively, on the apoplastic pathway, NPs of larger sizes (approximately 200 nm) are able to penetrate cell wall pores and then get through the space between cells (intercellular space) [136,137,138]. Leave uptake and translocation is believed to occur in two pathways: the cuticula pathway, where NPs penetrate through the cuticula, and the stomatal pathways, which involves NPs getting through stomatal openings [136,139,140]. Once the NPs are internalized, xylem and phloem pathways can transport NPs further away to other sections [136]. 

Hydroponic rice cultures treated during 14 days with TiO_2_ NPs at concentrations of 110 mg∙L^−1^, 250 mg∙L^−1^, and 500 mg∙L^−1^ caused biomass and antioxidant defense reduction [141]. Furthermore, an increase in the concentration of glucose-6-phospahte, glucose-1-posphate, succinic, and isocitric acid was found, while sucrose, isomaltulose, and glyoxylic acid concentration was decreased. According to the research of Al–Oubaidi [142], the TiO_2_ NPs showed an increase in phenolic and flavonoids compounds in *Cicer arietnum* when exposed to concentrations of 0.5 mg∙L^−1^, 1.5 mg∙L^−1^, 3 mg∙L^−1^, 4 mg∙L^−1^, and 5 mg∙L^−1^ [142]. 

Secondary metabolites can be increased deliberately to obtain a certain kind of product useful to human beings, mainly in medicinal plants. *Stevia rebudiana* plants treated with CuO and ZnO showed that the presence of these NPs generates toxic free radicals, and an increase of the concentration of these NPs results in the increase of the stress levels, enhancing all antioxidant activities. In conclusion, these NPs can be used as abiotic elicitors to produce plants with high antioxidants content [143]. 

Even though the size of the plant is enlarged by the presence of NPs, some results have found a decrease in total biomass. Arabidopsis thaliana in contact with TiO_2_ (100–1000 mg∙L^−1^) showed an enhancement in root growth but a decrease in total biomass and chlorophyll content as the concentration of TiO_2_ increases. High concentrations of TiO_2_ NPs cause lipid peroxidation, affecting antioxidant response and altering biosynthetic genes, which cause changes in vitamin E content [144].

Some types of metal oxide NPs found application in agriculture by promoting plant growth. ZnO NPs synthesized by green methods with an average size of 35 nm showed that when applied to wheat crops at concentrations of 15, 62, 125, 250, and 500 mg∙L^−1^, better growth is observed than that with control seeds; the root and shoot length showed significant enhancement, suggesting that ZnO NPs can be an ideal source of Zn micronutrients to the wheat plant development [145].

Spray treatment of Fe_2_O_3_ and ZnO on wheat showed accelerated plant height, leaf area, and shoot dry weight with no negative effects on chlorophyll content; also, leaf Zn and Fe content were increased with the spray treatment of NPs [146], while α-Fe_2_O_3_ in contact with *Citrus maxima* showed accumulation by plant roots and a decrease in chlorophyll content, implying chloroplast sensitivity toward iron oxide NPs [147].

As discussed above, changes in nanoparticle characteristics (morphology, concentration, size distribution, etc.) can lead to different results. For example, the toxicological effects of uncoated and citric acid-coated cerium oxide NPs were studied on tomato plants [148], showing that at 500 mg∙kg^−1^, both types of NPs increased shoot length, while bulk cerium and acid-coated cerium NPs cause a shoot length decrease and an increase in catalase activity.

The physicochemical properties of the NPs are vital for understanding their interaction, uptake, and distribution along with the plant system. Since every plant species has different anatomy, the toxicological data can be complicated to study; even though some studies try to explain the biodistribution and uptake of NPs along different plants, more studies and research are needed [149]. Plant toxicity is affected by the general characteristic of the NPs (size, morphology, type of coating in some cases, concentration, electrical charge, crystal structure, etc.), type of application, and the applied experimental method, as shown in Table 3 [150].

##### Carbon Based Nanomaterials

Some studies find carbon-based nanomaterials as ideal products for increasing plant yield quality as fertilizers, products for protecting plants such as pesticides and herbicides. However, their interaction and effects will depend on the plant and the nanomaterial characteristics. Carbon-based nanomaterials can increase the formation of reactive oxygen species [33]; also, they have the ability to get through various types of cells depending on the size. Some studies claim that carbon nanotubes larger than 200 nm accumulate in subcellular organelles, while smaller nanotubes (30–100 nm) can penetrate the vacuole and nucleus [171] as well as have effects on the soil bacterial community [172]. 

Rice plants exposed to several carbon nanomaterials (nanotubes, C_60_, graphene) showed that those materials have the ability to increase water content in seeds and also have the ability to be upward translocated to leaves [173]. Enhanced water uptake by carbon nanomaterials was also found in *Cicer arietinum* treated with water-soluble carbon nanotubes (wsCNTs) enhancing the root, shoot, and branching growth rate. The increase in water retention could be due to the attachment of CNTs to root surfaces or inner parts such as vascular bundles. The alignment of the nanomaterial is believed to enhance the capillarity absorption of water; also, it is suggested that CNTs serve as tubular membranes for molecular transport [174]. Table 4 shows more studies of carbon-based nanomaterials interacting with plants.

As can be noticed, the use of diverse NPs as stress promoters in plants shows some effects related to the growth, biomass yield, and effects over the self-defense mechanisms of the plant. These self-defense mechanisms such as the production or inhibition of secondary metabolites by the reactive oxygen species (ROS) production may point to the optimal conditions according to the NPs application dose or exposure to the plants. In addition, NPs characteristics are crucial to obtain crops with maximized quality and nutrient content.

To assure the preservation and reproduction of plants, the primary metabolism plays a major role; it is involved in growth and energy production processes. It is characterized by the production of carbohydrates, proteins, and fatty acids, which are required for plant nutrition and sustaining primary and secondary metabolism [189].

The presence of secondary metabolites is related to external changes, environmental conditions, physical and chemical attacks, or even competitor organisms that can trigger the production of these compounds in plant tissue [190,191]. 

Primary and secondary metabolites can be produced in different plant sections, such as leaves, roots, and the stem, with varying concentrations at distinct stages of plant development, such as seedling, maturity, and fruit/flower production [104]. Secondary metabolites are the chemical compounds responsible for the medicinal value of some plants [192]. These compounds are in concentrations of parts per million (ppm), so to increase the total content of these secondary metabolites in the plant, several molecules called elicitors are used to activate the plant defense system and force it to produce the desired secondary metabolites, which are also called phytohormones or phytochemicals [193]. Although the main use for these phytochemicals is for medicinal purposes, they are used as flavorings, agrochemicals, fragrances, colors, biopesticides, and food additives [193].

The presence of secondary metabolites into plants can fluctuate according to the types and level of the applied stress, such as drought, salinity, temperature, biotic factors (pests, competitive species, fungus, viruses). In addition, factors such as radiation, chemical stress, which includes NPs, seasonal variation, and region/location can also affect plant metabolism [194]. 

Many secondary metabolites have very interesting applications in fields such as dyes, cosmetics, flavoring, odor, nutraceuticals, and medicinal preparations. Although their presence in plants are in low concentrations, stress-inducing agents can be used to activate plant defense mechanism to trigger and increase the secondary metabolites production [102].

## 6. Plant Elicitors 

The stress-inducing agents are called elicitors. The elicitors can be classified as biotic (fungal homogenates such as *Phytophthora*, *Aspergillus*, and *Alternaria*, insects, microorganisms) and abiotic elicitors (salinity, temperature, light, wounds, and metallic ions, among others). Recently, it was found that several NPs can also act as elicitors, forcing the plant to defend itself against them, and in consequence, the plant produces the desired metabolite [190].

### 6.1. Nanoparticle Elicitors 

#### 6.1.1. Metallic NPs

Cobalt NPs with 10 nm of diameter have shown a potential application for enhancing artemisinin (medicine compound) content at low concentrations, where 5 mg∙L^−1^ of cobalt NPs caused an inhibition of certain genes in *Artemisia annua* suspension cultures, which caused the increase of artemisinin content [195]. An improvement of the medicinal qualities in *Calendula officinalis* L. was also achieved using silver NPs in combination with methyl jasmonate, where both compounds increased saponin content by 177% compared to control, while all treatment reduced anthocyanin and flavonoid content [196].

A comparative study between Ag NPs and Ag^+^ ions toward metabolism in *Arabidopsis thaliana* where a hydroponic exposure of NPs at 1.0 and 2.5 mg∙L^−1^ affected more the plant shoot and root growth compared to the ions, a stronger stimulation of energy metabolic pathways such as trucarboxylic acid cycle and sugar was achieved by NPs causing an accelerated metabolic response toward silver NPs, which led to reduce plant yield [197].

Metal alloy NPs and their plant interaction need more research to comprehend their effects. *Silybum marianum* was treated with several monometallic and metallic alloys such as (Ag, Au, Cu, Ag–Cu, Au–Cu, and Ag–Au) at different ratios, where all NPs increased germination frequency, shoot and root development at different ratios depending on the nanoparticle. In addition, Ag–Cu and Ag–Au alloys were able to increment phenolic content, while other NPs treatments were able to also increase flavonoid content [198]. Table 5 shows multiple types of research with metallic NPs modifying the secondary metabolites contents in several plant species. 

#### 6.1.2. Metal Oxide NPs

Metal oxide NPs can act as elicitors; the nanoparticle characteristics and interaction between NPs and plants discussed in the previous sections are key facts that can generate different results in plant elicitation. Oxide NPs affect antioxidant enzymes such as super superoxide dismutase (SOD), peroxidase (POX), and catalase (CAT), while also stimulating the production of bioactive compounds [206].

Titanium dioxide showed an elicitation of secondary metabolism in *Salvia officinalis* obtaining the highest phenolic and flavonoid contents at 200 and 100 mg∙L^−1^, respectively. Monoterpenes (mayor component in essential oils) were increased in plants exposed to 200 mg∙L^−1^ of TiO_2_ NPs. Monoterpenes increase could be considered as a defense mechanism against free radicals generated by the NPs [207]. Meanwhile, *Mentha piperita* L. treated with 150 mg∙L^−1^ of TiO_2_ NPs also showed an increase in essential oil contents by 105.1% compared to control. Increased essential oil content can be due to the elicitor effect of TiO_2_, through jasmonic acid and methyl-ester signaling. The menthol content increase in *Mentha piperita* L. can be due to an increased expression of reductase enzyme by the NPs [208].

*Stevia rebaudiana* callus was treated with ZnO NPs, where plants treated with 100 mg∙L^−1^ showed increased phenolic and flavonoid content. ZnO NPs generate reactive oxygen species enhancing antioxidant responses [143]; also, ZnO NPs with 34 nm in size (1 mg∙L^−1^) almost doubled the steviol glycosides content in *Stevia rebaudiana*, while the secondary metabolites content showed a decline after crossing 1 mg∙L^−1^ [186]. The germination stage can also be promoted via nanoparticle interaction; *Capsicum annuum L*. in contact with ZnO NPs showed improved seed germination, increasing seed vigor up to 123.50%, 129.40%, and 94.17% at 100 ppm, 200 ppm, and 500 ppm, respectively. In addition, there was an increased phenolic compounds concentration and antioxidant activity on seedling radicles due to a phytotoxic effect caused by ROS [209].

Airborne NPs also play an important role in ecology safety. Barley plants were treated with cadmium oxide (CdO) NPs (7–60 nm) in air. CdO NPs were directly absorbed by leaves, inducing changes in the primary metabolites content—mainly amino acids and saccharides (tryptophan and phenylalanine), while the secondary metabolites content remained unchanged [210]. 

The production of bioactive compounds can be achieved through hairy root cultures, CuO NPs elicited Chinese cabbage hairy roots, highly increasing the total phenolic and flavonoid contents, showing an efficient technique for nutraceutical uses [211]. The toxicological effects of CuO NPs were studied in *Brassica rapa* seedlings, and CuO NP-treated showed enhanced reactive oxygen species and hydrogen peroxide production, which could have caused DNA damage. Due to NPs stress, glucosinolate, and phenolic compounds content were significantly increased in the seeding process, while the chlorophyll, carotenoid, and sugar content decreased [211]. 

Mn_2_O_3_ NPs of 30 nm were tested in Muraishige and Skoog (MS) culture medium with concentrations of 25 mg∙L^−1^, 50 mg∙L^−1^, 100 mg∙L^−1^, and 200 mg∙L^−1^, showing morphological changes in root and shoot fresh weight. Physiological parameters such as chlorophyll and hydrogen peroxide content at low concentrations of the NPs showed enhance growth and increase in the secondary metabolites synthesis (alkaloids, phenolics, and flavonoids) [212]. Table 6 shows multiple types of research with metal oxides modifying the secondary metabolites contents in several plant species.

#### 6.1.3. Carbon-Based Nanomaterials

Although some studies claim that carbon-based nanomaterials have potential applications in agriculture, some experiments have shown hazardous effects in several plant species, plant growth, oxidative stress, water channels, secondary and primary metabolism, and gene expression [236]. Graphene in contact with wheat (*Triticum aestivum* L.) at low concentrations (250 mg∙L^−1^) caused root elongation. As claimed by other studies, it is believed that root elongation is caused by an elongation of cell walls when interacting with NPs. Even though root elongation was achieved, an exposure to 500 mg∙L^−1^ causes short root hairs as compared to control, which is believed to be an effect of ROS interactions. Meanwhile, lower concentrations of graphene caused SOD and POD bursts, while higher concentrations caused an inhibition of these enzymes [237]. This is known as a hormesis effect, where low doses of certain compounds cause toxic effects, while higher doses decrease those effects [238,239,240]. 

In case of carbon nanodots, it was found that they can be used as a highly efficient defense system against abiotic stresses, improving crop performance. Rice (*Oryza Sativa* L.) showed an alleviation of salt stress when incubated with CDs. The alleviation is believed to be caused by the ROS scavenging properties of QDs lowering the exposure of these radicals to seedlings and minimizing oxidative damage, which was related to a lower accumulation of POS, SOD, flavonoids, and phenols content compared to control [241].

One important aspect to consider is the interaction between nanomaterials and other contaminants such as heavy metals. Some of them have the ability to sequester heavy metals to remediate soil and protect the plant, but it can be possible that some NPs increase the toxic effect of certain compounds. As it is shown, in wheat plants treated with graphene oxide (GO), the nanomaterial caused cellular damage, augmenting arsenic uptake. Both compounds caused an inhibition of carbohydrates and disrupted fatty acids. While enhancing amino acid and secondary metabolism, GO also caused the reduction of As(V) to As(III), increasing its toxicity [242].

Fullerol C_60_(OH)_20_ showed an increase in plant biomass and fruit yield when exposed to bitter melon (*Momordica charantia*). In addition, it also increased its medical content of two known antidiabetic compounds (charantin and insulin). Even though some carbon-based nanomaterials can increase water uptake, fullerol caused no impact in water uptake [243].

Another structure of carbon-based nanomaterials are single-walled carbon nano-horns, which have been tested in several crops such as barley, corn, rice soybean, switchgrass, tomato, and a tobacco cell culture. The findings elucidate that carbon nano-horns can increase the germination of selected species and increase organ growth in corn, tomato, and soybean. Meanwhile, tobacco cell culture showed increase growth levels [244].

Foliar exposure to multi-walled CNTs on *Salvia verticillara L*., a medicinal plant, showed increased levels of oxidative stress in leaves, as well as decreasing photosynthetic pigments. CNTs also increased rosmarinic acid, leading to future applications for enhancing pharmaceutical metabolites contents by carbon nanomaterials [245]. Table 7 contains multiple research studies showing metabolomics modifications by carbon nanomaterials.

## 7. Conclusions

Nanotechnology is an effective tool for the improvement of the agricultural industry. However, it expresses different behaviors to their counterparts in bulk in such a way that the nanotoxicological effects are based on totally different parameters that are not based on the number of doses with respect to the mass. Instead, characteristics such as the aggregation, morphology, concentration, surface modification, and size define the level of toxicity of nanomaterials, and in turn, the possible diverse biochemical effects they can cause in plants. 

Regardless of its origin either as a product with a specific purpose for agriculture or its possible introduction to the environment through the mishandling of wastes that contain nanomaterials, it is imperative to know for sure the toxicological effects that nanostructured systems can cause on plant organisms. Thus, it is crucial to create strict and efficient regulations with which their misuse can be prevented by protecting other plant species whose interactions with certain nanomaterials generate highly adverse effects for their development.

The secondary metabolites when discovered were considered low-interest compounds. However, they are products that over time have increased their value for commercial use in dyes, fragrances, flavorings, and even bioactive compounds for medical areas. Stressful environmental factors such as temperature, radiation, salinity, droughts, floods, the presence of insects, microorganisms, and viruses have shown the mechanisms by which plants can increase the synthesis of the so-called secondary metabolites or phytochemicals. The use of elicitors increases the global production of phytochemicals; thus, we consider that this is an area with high opportunities where the toxicological effects of diverse nanomaterials can be exploited for the induction of plant stress, leading to secondary metabolites productions that can surpass the commonly used elicitors.

## Figures and Tables

**Table 1:** Agricultural applications of nanoparticles (NPs).

Nanoparticle	Agricultural Use	Ref
Metal oxide NPs		
ZnO	Remediation and fortification of rice with low concentrations of Zn in soil.	[50]
MnO_x_	Colorimetric nanosensor for indirect measurement of antioxidant capacity.	[67]
CuO, ZnO	Both NPs facilitate bifenthrin insecticide uptake in *Eisenia fetida* earthworms compared to only bifenthrim exposure.	[68]
MnO	Antifungal activity against soil-borne pathogens (*P. nicotianae, T. basicola*) with possibility to control other plant pathogens.	[69]
ZnO quantum dot	Sensor for pesticide detection in water.	[70]
Zeolite/Fe_2_O_3_	Nanofertilizer with less toxic effect toward humans compared to other fertilizers.	[71]
SiO_2_	Insecticide properties against leaf worm (*Spodoptera littoralis*).	[72]
Yb_2_O_3_	Fluorescent sensor for imazapyr herbicide detection.	[73]
TiO_2_	Antifungal activity against wheat rust.	[74]
ZnO nanobuboids	Pt/ZnO/AChE/Chitosan bioelectrode for sensing carbosulfan pesticide in rice.	[75]
CuO	Biosensor for detection of *Aspergillus niger* fungus.	[76]
ZnO	Fungicidal activity against multiple pathogenic fungi of apple orchards (*Alternaria mali, Botryosphaeria dothidea, Diplodia seriata*).	[77]
Cu-TiO_2_	Electrochemical sensor for the selective detection of methyl parathion pesticide.	[78]
ZnO	NPs enhances thiamethoxam insecticidal activity against *Spodoptera litura* larvae.	[79]
SnO_2_/Pd	Nanosensor for the detection of fungal volatile organic compounds.	[80]
Urea-loaded mesoporous ZnAl_2_Si_10_O_24_	Nanofertilizer for slow delivery of urea and zinc.	[81]
ZnO	Nanofertilizer with capacity to reduce arsenic and cadmium contents in rice cultures.	[82]
CuO	Antifungal activity against plant pathogen *Colletotrichum gloeoesporioides*	[83]
SiO_2_	Maize nanofertilizer and low-dose pesticide for pest that can affect maize during storage post-harvest (*Sitophilus oryzae, Rhizopertha dominica, Tribolium castaneum, Orizaephilus surinamenisis*).	[84]
Metallic NPs		
Cu nanowires	Fertilizer for improved plant physiological performance and agronomical parameters.	[85]
Cu NPs	Metal NPs for synergic action with conventional fungicides, reducing fungicide uses.	[86]
Zn and Cu	NPs for increased quantity and quality in basil.	[87]
Ag	Fungicidal activity against agricultural pathogens.	[88,89]
Au	NPs for colorimetric detection of organophosphorus pesticides.	[90]
Organic NPs		
Ag@Chitosan	Synergetic antifungal activity with Antracol fungicide against *Phytophthora capsici*.	[91]
Chitosan with Cu and salicylic acid	Nanofertilizer for obtaining higher crop yield.	[92]
Alginate/chitosan Chitosan/tripolyphosphate	Nanocarrier for planth growth regulators (gibberellic acid) release.	[93]
Zn–Chitosan	Complex for crop Zn biofortinification.	[94]
Carbon Nanomaterials		
CNT–NH_2_	Biosensor for organophosphorus pesticide detection.	[95]
Ionic liquid polymer functionalized CNTs-doped poly(3,4-ethylenedioxythiophene)	Coating for high selective extraction of carbamate pesticides	[96]
C_60_-L-Threoninde	NPs for decreasing pesticide load on the environment with plant growth-stimulating abilities.	[97]
Graphene oxide–Fe_3_O_4_	Antifungal agent against *Plamopara viticola*.	[98]
Carbon dots	Agent for increasing growth and photosynthesis.	[99]
Graphene/Fe_3_O_4_	Agent for fungicide removal of triazole fungicides.	[100]

**Table 2:** Effects detected by the interaction of metallic NPs over different crops.

NPs	Concentration	Plant	Effect	Ref
Ag	10, 100, 1000 mg∙L^−1^	Lettuce (*Lactuca sativa*)	No sign of phytotoxic effect on foliar application, Ag NPs were trapped in lettuce leaves.	[124]
Au	31.25 nM	Rice, perennial ryegrass, radish, and pumpkin	Positive charged NPs are taken by the root, while negative charged are easily translocated into shoots.	[125]
Ag	0, 5, 10, 20, and 40 mg∙L^−1^	Mung bean (*Phaseolus radiates*) and sorghum (*Sorghum bicolor*)	Seedling growth affected by NPs, growth rate of P. radiatus not affected by Ag in soil media.	[126]
Phytochemical capped Au NPs	5–15 mg∙L^−1^	Maize	Boosted germination of aged maize seed.	[127]
Ag	10, 100, 200, 500, 1000, 2000 mg∙L^−1^	Carrot (*Daucus carota* L.)	Reduced germination rate, seed growth, seed protein, increased chlorophyll, and H_2_O_2_ content.	[128]
Ag and Au	10 and 30 mg∙L^−1^	Chrysanthemum, gerbera, and cape primrose	Ag inhibits rhizogenesis in chrysanthemum and gerbera, Au enhances root regeneration (gerbera), while both NPs increase cape primrose micropropagation.	[129]
Au	10 μg∙L^−1^	*Arabidopsis thaliana*	NPs enhance seed yield, germination rate, growth, and radical scavenging activity.	[130]
Au and Ag	5.4 mg∙L^−1^	Onion (*Allium cepa* L.)	Au at 5.4 ppm enhances germination, plant height, leaf length, leaf diameter without toxicity symptoms; Ag improved onion seeds germination.	[131]
Au	0, 10, 25, 50 and 100 mg∙L^−1^	*Brassica juncea*	Foliar spray increases plant height, stem diameter, seed yield (10 ppm), and reduced sugar content (25 ppm).	[132]
Fe, Cu, Ni	0.0125 to 1.0 M	*Triticum vulgare* L.	Fe NPs stimulated growth compared to control, Ni and Cu NPs caused toxic effects on growth as metal content elevated, they also caused at low concentrations root growth reduction.	[133]

**Table 3:** Effects detected by the interaction of metal oxide NPs over different crops.

NPs	Concentration	Plant	Effect	Ref.
ZnO, CuO and CeO_2_	100, 500, and 1000 mg∙kg DW^−1^	Sweet potato	Yield affected at high concentrations	[151]
CuO	0.1, 1.0, and 10.0 g∙L^−1^	Duckweed	Changes in, growth rate, and photosynthetic content	[152]
Fe_2_O_3_	20, 50, and 100 mg∙L^−1^	Maize	Decrease in root length at concentrations of 50 and 100 mgL^−1^	[153]
TiO_2_	10 and 40 mg∙L^−1^	Dragonhead	Increase in plant shoot and essential oil content	[154]
ZnO	2000 mg∙L^−1^	Maize and rice	Root elongation significantly decreased	[155]
TiO_2_	1000 mg∙L^−1^	Wheat	Early growth parameter adversely affected	[156]
CeO_2_	1000 and 2000 mg∙kg^−1^	Romaine lettuce	Lower chlorophyll content and biomass production	[157]
TiO_2_	100–1000 mg∙L^−1^	Thale cress	Chlorophyll content reduction, plant biomass modification, and antioxidant enzymes alteration	[144]
CeO_2_	250–500 mg∙kg^−1^	Tomato	Increase in shoot length and chlorophyll content	[148]
CoFe_2_O_4_	1000 mg∙L^−1^	Tomato	No effects on germination, root length improved	[6]
NiO_2_	120 mg∙kg^−1^	Wheat	Reduction in plant growth, increase in antioxidant content and photosynthesis inhibition	[158]
ZnO	15, 62, 125, 250, and 500 mg∙L^−1^	Wheat	Enhancement in root and shoot length	[145]
CuO	5, 10, and 20 mg∙L^−1^	Alfalfa and Lettuce	Decrease in root and shoot length, modification in enzyme activity	[159]
Al_2_O_3_	2000 mg∙L^−1^	Maize	Slightly toxic to root elongation	[155]
TiO_2_	10 mg∙L^−1^	Mug bean	Modification in shoot length, root length, chlorophyll content, and total soluble leaf protein	[160]
CuO	10 mg∙L^−1^	Thale cress	Root damage	[161]
CuO	500–1000 mg∙L^−1^	Watermelon	CuO NPs increased biomass and produce more fruit than untreated controls	[162]
γ-Fe_2_O_3_	100, 250, 500, 1000, and 2000 mg∙L^−1^	Maize (*Zea mays L*) and rice (*Oryza sativa*)	γ-Fe_2_O_3_ caused the highest seed germination percentage and seedling vigor index at 500 ppm for both crops	[163]
TiO_2_, SiO_2_	1000 mg∙L^−1^	Maize seedlings (*Zea mays* L.)	SiO_2_ reduced shoot length and shoot fresh weight; TiO_2_ caused a pigment content reduction	[164]
TiO_2_	30, 50, and 100 mg∙kg^−1^	Wheat (Triticum aestivum)	NPs enhanced root and shoot length and nutrient content in shoots (Ca, Cu, Al, Mg), crude protein content enhanced with 50 mg∙L^−1^ exposure	[165]
Fe_2_O_3_	500 mg∙kg^−–1^	Wheat	Fe_2_O_3_ enhanced root length, plant height, biomass, and chlorophyll content, NPs were translocated to the leaves and caused root tip damage	[166]
CuO	0.2–300 μg∙mL^−1^	Lettuce (*Lactuca sativa* L.)	Inhibition of seed germination and radicle growth (40 μg∙mL^−1^); S-nitrosothiols levels in radicles showed direct dose–response to NPs.	[167]
Al_2_O_3_	0.4, 1, and 2 mg∙L^−1^	Lettuce (*Lactuca sativa* L.)	NPs absorbed by root promoted macronutrient uptake, adsorption, and aggregation of NPs limited translocation to root	[168]
Al_2_O_3_	1.25 to 5 μM	*Allium cepa*	Micronuclei and DNA damage with an increase in concertation	[169]
TiO_2_, Fe_2_O_3_, CuO	50 and 500 mg∙kg^−1^	Wheat (*Triticum aestivum*)	Fe, Zn, and essential amino acid content decrease with CuO application, TiO_2_ increased amino acid accumulation, Fe_2_O_3_ increase cysteine and threonine contents	[170]

**Table 4:** Effects detected by the interaction of carbon-based nanomaterials over different crops.

NPs	Concentration	Plant	Effect	Ref
Mesoporous carbon	0, 10, 50, and 150 mg∙L^−1^	Rice (*Oryza sativa* L.)	Decrease in root and shoot length (150 mg∙L^−1^) and increase of phytohormones.	[175]
Multiwalled CNTs	10, 100, 200, 500, 1000, and 2000 mg∙L^−1^	Carrot (*Daucus carota* L.)	No change in seed germination, decrease in in seed protein level and H_2_O_2_ content, increase in chlorophyll content (500 mg∙L^−1^).	[128]
C_60_ and salicylic acid	0, 125, 250, 500, and 1000 mg∙L^−1^	Feverfew (*Tanacetum patthenium* L.)	Improved growth at higher concentrations, the maximum increase of flower at 1000 mg∙L^−1^, increase in chlorophyll content at low C_60_ levels.	[176]
Graphene	500–2000 mg∙L^−1^	Cabbage, tomato, red spinach, lettuce	Plant growth and biomass inhibition, dose-dependent reduction of leaves number, reactive oxygen species (ROS), and cell damage increase; no significant toxic levels were found in lettuce.	[177]
Graphene oxide (GO), GO quantum dots, and reduced GO (rGO)	0.5, 5, and 50 mg∙kg^−1^	Wheat	Decreased mineral elements, upregulation of sugar content, rGO downregulates proteins and reduces globulin, prolamin, amylose, and amylopectin.	[178]
Graphene oxide	0, 30, and 60 mg∙L^−1^	Lettuce	Increased total length, hair root numbers (30 mg∙L^−1^), foliar application improved quality of lettuce, increase in sugars, proteins, and vitamin C (30 mg∙L^−1^).	[179]
Graphene nanosheets	0.1, 0.2, and 0.3 g∙L^−1^	Pepper, (*Capsicum annuum* L.), eggplant, (*Solanum melongena* L.)	Improvement plant yield and growth, no membrane damage detected, nanosheets located inside the chloroplast, stimulation of sugars, and rise of H_2_O_2_.	[180]
Multi-walled CNTs	0, 25, 50, and 100 mg∙L^−1^	*Sweet basil* *Ocimum basilicum* L.	NPs induces plant growth and elevates essential oil content, high dosages (100 mg∙L^−1^) lead to toxicity in plant tissue.	[181]
Multi-walled CNT-carboxylic acid functionalized Single-wall CNTs (SWNT)	1 and 10 mg∙kg^−1^	Tomato	Carbon nanotubes (CNTs) did not affect plant growth and height, SWNT increases salicylic acid content.	[182]
MWCNTs	0, 125, 250, and 500 μg∙mL^−1^	*Cucurbita pepo* L.	Reduction of germination percentage, shoot length, biomass, increase in oxidative damage.	[183]
CNTs	0.01 to 1000 g per ton of seeds	White mustard	Germination energy and viability inhibited by all concentrations except 0.01 g∙t^−1^.	[184]
MWCNTs	0 and 500 mg∙L^−1^	Onion (*Allium cepa* L.)	Increase levels of plant height, chlorophyll rate, and leaf area.	[185]
ZnO/MWCNTs	0, 2, 5, 10, 15, 20, and 40 μg∙mL^−1^	Onion (*Allium cepa* L.)	Enhanced seedling growth.	[186]
Carbon dots	0, 10, 20, and 30 mg∙L^−1^	*Lactuca sativa* L.	Increase production yield, growth rate, and decrease of nitrates content.	[187]
MWCNTs	0, 10, and 50 mg∙L^−1^	Maize and soybean	MWCNTs accumulated in xylem and phloem, stimulation of growth in maize and growth inhibition in soybean was observed, dry biomass of treated maize was higher than control.	[188]

**Table 5:** Multiple findings toward metabolomics effects in plant due to metallic NPs treatment.

NPs	Plant Type	Nanoparticle Characteristics	Experiential Conditions	Effects	Ref
Ag	Rice	Size 18.6 nm	0, 10, 20, and 40 mg∙L^−1^	Increased content of chlorophyll a and carotenoid content, elevation of catalase (CAT), APX, and GR activity.	[199]
Ag	*Arabidopsis*	Triangular (47 ± 7 nm), spherical (8 ± 2 nm), decahedral (45 ± 5 nm)	100 μM	Spherical NPs enhanced anthocyanin accumulation in seedlings; the three morphologies induce protein accumulation.	[200]
Ag	Fenugreek *(Trigonella foenum-graecum)*	Synthetized by reduction of silver nitrate.	0, 20, 40, and 60 mg∙L^−1^	Improved shoot length, leaves, and plant number, an increase of photosynthetic pigments (chlorophyll and carotenoids), phenolics, flavonoids, and tannins.	[201]
Ag	Wheat and tomato	17 nm	100 mg∙L^−1^ (10 days exposure)	Ag NPs had no significant effect on germination, and pigment content on wheat and tomato exposed to Ag NPs caused a reduction in chlorophyll.	[202]
Ag	Cucumber (*Cucumis sativus*)	Size: 20 nm.	Foliar application (4 and 40 mg/plant)	NPs caused an activation of antioxidant defense, upregulation of phenolic properties, and altered membrane properties.	[203]
Ni	*Triticum aestivum* L.	5 nm	0.01, 0.1, 1, and 10 mg∙L^−1^	Suppression of root growth at 1 and 10 mg∙L^−1^, the content of chlorophyll decreased due to NPs, carotenoids content decreased in a dose-dependent manner, flavonoid content also decreased.	[204]
Ni	*Corianderum sativum* L.	20 nm	20, 40, and 80 mg∙L^−1^	NPs decrease relative to water content, photosynthetic pigments, root and shoot elongation, antioxidant activity decreased in a concentration-dependent manner.	[205]

**Table 6:** Multiple findings toward metabolomic effects in plant due to metal oxide NPs treatment.

NPs	Plant Type	Nanoparticle Characteristics	Experiential Conditions	Effects	Ref.
TiO_2,_ ZnO	Beetroot	≤ 40 nm size	Culture cell (0.25, 0.50 ml∙L^−1^ NPs).	ZnO and TiO_2_ improved chlorophyll content, plant growth, and carotenoid (terpenes) content.	[213]
Fe_3_O_4_, CuO	*Lepidium draba*	Particle size of 60 nm and 55 m^2^g^−1^ surface area	Seed culture (0, 1, 5, 10, 20, and 40 mg∙L^−1^).	CAT and POD activity enhanced by both NPs, increased concentration of sulforaphane.	[214]
Fe_3_O_4_	*Hyoscyamus reticulatus* L.	Nanoparticle solution provide by Nanozaino Co., Tehran, Iran.	Hairy root culture with different concentrations (0, 450, 900, 1800, and 3600 mg∙L^−1^).	Antioxidant enzyme activity increased, hyoscyamine and scopolamine elicited by NPs.	[215]
CeO_2_	*Phaseolus vulgaris var.*	CeO_2_ rods 67*8 nm, 93.8 m^2^g^−1^ surface area.	Root exposure with NPs suspensions of 62.5, 125, 250, and 500 mg∙L^−1^.	Increase in soluble protein content by 204% at 500 mg∙L^−1^.	[216]
ZnO, Fe_3_O_4_	*Hypericum perforatum.*	Nanoparticle powder obtained from Plasma chem, Germany.	Cell culture with concentrations of (0, 50, 100, and 150 ppb).	Enhanced production of hypericin and hyperforin.	[217]
CuO	*Stevia rebaudiana.*	40–100 nm synthesided by co-precipitation method.	Murashige and Skoog medium (MS) (0, 0.1, 1, 10, 100, and 1000 mg∙L^−1^).	CuO oxidative stress activates the production of antioxidative molecules (phenols, flavonoids), CuO also enhanced rebaudioside A and stevioside (steviol glycosides) production.	[218]
ZnO-polyethylene gycol (PEG), ZnO-polyvinyl pyrrolidone (PVP), CuO-PEG and CuO-PVP and CuO, ZnO.	*Stevia rebaudiana*	ZnO (34 nm), ZnO–PEG (26 nm), ZnO–PVP (32 nm), CuO (47 nm), CuO–PEG (27 nm), CuO–PVP (27 nm), synthetized by chemical co-precipitation.	Murashige and Skoog medium (1 and 10 mg∙L^−1^).	Metal oxide NPs capped with polymers resulted in larger steviol glycosides content, total phenolic content, and total flavonoid content compared with uncapped metal oxide NPs.	[219]
Ag–SiO_2_	*Artemisia annua.*	Core–shell structure (101.8 nm)	Hary root cultures (400, 900, 1800, and 3600 mg∙L^−1^).	Increased artemisinin content, enhaced activities of catalase (CAT).	[220]
TiO_2_	*Salvia officinalis.*	TiO_2_ anatase (10–15 nm), 200–240 m^2^∙g^−1^ surface area.	Solution sprayed to plants (0, 10, 50, 100, 200, and 1000 mg∙L^−1^).	Treated plants showed increased antioxidant activity, the highest concentrations of phenols and flavonoids were observed at 200 and 100 mg∙L^−1^.	[207]
CuO	*Brassica rapa* spp. pekinensis	25–55 nm particle size.	Hairy root cultures (0, 50, 100, and 250 mg∙L^−1^)	CuO elicited glucosinolates content; also, phenolic compounds were highly enriched.	[211]
Bulk and nano TiO_2_	*Hyoscyamus niger* L.	10–15 nm, 200–240 m^2^∙g^−1^.	Solution sprayed to plants (0, 20, 40, and 80 mg∙L^−1^)	Increases superoxide dismutase (SOD) by nano and bulk TiO_2_, highest alkaloid (hyoscyamine and scopolamine) content registered in nano TiO_2_ at 80 and 20 mg∙L^−1^.	[206]
Fe_2_O_3_Fulvic acid coated Fe_e_O_3_, Fe-EDTA	Soybean *(Glycine max* L.*)*	5 nm particle size	Foliar and soil exposure to NPs (15, 30, and 60 mg/pot) for eight weeks	No stress and growth disorders, Fe_2_O_3_ and fulvic acid-coated enhanced chlorophyll content, plant biomass, and root development.	[221]
γ-Fe_3_O_3_Fe_3_O_4_	Muskmelon *(Cucumis melo)*	γ-Fe_3_O_3_ (20 nm) Fe_3_O_4_ (20 nm)	Soil irrigation (100, 200, and 400 mg∙L^−1^) for 4 weeks	Increase chlorophyll and fruit weight at concentrations of 200 mg∙L^−1^ for both types of NPs.	[222]
TiO_2_	Wheat *(Triticum aestivum)*	Anatase/rutile mixture (80:20); 21 nm and 35.65 m^2^∙g^−1^ surface area	Soil irrigation (5, 50, and 150 mg∙L^−1^) for 21 days.	During treatment, roots upregulated monosaccharides and azelaic acid, triggering tyrosine metabolism; leaves showed upregulation of reserve sugars and tocopherol, phenulalanine, and tryptophan pathways.	[223]
CuO	Rice *(Oryza sativa* L.*)*	Size ranging from 40 to 80 nm	Hydroponic treatment (62.5, 125, and 250 mg∙L^−1^).	Suppression of growth rate of rice seedlings; chlorophyll and carotenoid content in leaves decreased with NPs exposure.	[224]
TiO_2_	Rice *(Oryza sativa* L.*)*	Anatase (5–10 nm)	Soil exposure to NPs (0.1–100 mg∙L^−1^).	Increased biomass (>30%), the photosynthetic rate decreased at 10 and mg∙L^−1^ (17.2%), NPs caused a downregulation of energy consumption in metabolism.	[225]
Fe_3_O_4_	Maize *(Zea mays)*	30 nm NPs, hydrodynamic diameter of 231.4 ± 17.38 nm, z potential of 17.97 mV	Soil exposure during 4-week treatment (50 and 500 mg∙kg^−1^).	No impact on biomass and photosynthesis, increased Fe accumulation in roots, metabolomics pathways related to defense were inactivated after NPs exposure.	[226]
Al_2_O_4_, NiO	*Nigella arvensis L*	NiO (5–8 nm) Al_2_O_4_ (5 nm)	Hydroponically grown tissues (50, 100, 1000, and 2500 mg∙L^−1^).	Plant biomass increased at 50 and 100 mg∙L^−1^ (Al_2_O_3_) and 50 mg∙L^−1^ (NiO), while higher concentrations decreased biomass; increase in antioxidant capacity, total saponin content, and total phenolic content in plants treated with 100–2500 mg∙L^−1^ of Al_2_O_3_.	[227]
Al_2_O_4_	*Arabidopsis thalian*	Al_2_O_3_ hydrodynamic diameter of 687.34 nm at 0 h, 878.82 nm at 12 h, and 908.97 nm at 24 h exposure	10-day exposure of 98 μM Al_2_O_3_.	No evidence of toxicity on photosynthesis, growth, and lipid peroxidation; NPs increased root weight, length, and the transcription of antioxidant-related genes.	[228]
NiO	Chinee cabbage	10–20 nm	50, 250, and 500 mg∙L^−1^.	Chlorophyll, carotenoid, and sugar contents were reduced, while proline and anthocyanins were upregulated in NiO NPs-treated seedlings.	[229]
TiO_2_	Radish *(Raphanus sativus* L.*)*	86 nm, zeta potential average of −7.0 mV, hydrodynamic diameter of 405 nm	Foliar application of NPs from 10 to 1500 mg∙L^−1^.	NPs caused an increase of photosynthesis and total phenols concentration, while higher doses of TiO_2_ contribute to instantaneous water-use efficiency.	[230]
Fe_3_O_4_	Pumpkin (*Cucurbita maxima* L.)	10–40 nm	Hydroponic treatment of pumpkin seedlings for 1 week (100 mg∙L^−1^).	Fe_3_O_4_ were found on pumpkin phloem sap revealing nanoparticle translocation; secondary metabolite analysis shows a reduction in the oil-related metabolites such as methoxyacetic acid, 4-tetradecyl ester eicosane, and heneicosane.	[231]
CeO_2_	*Bean* *(Phaseolus vulgaris* L.*)*	10−30 nm, surface area of 30–50 m^2^∙g^−1^	Plants grown in solid medium (25, 50, and 100 mg∙L^−1^).	Ce accumulated in roots and translocated to aerial parts, NPs caused tissue-specific metabolic reprogramming.	[232]
CuO	*Brassica rapa*	25–55 nm	Seedling grown in culture boxes (50, 250, and 500 mg∙L^−1^ of NPs).	Chlorophyll, carotenoid, and sugar content decreased; proline and anthocyanins were enhanced with CuO treatment; ROS, malondialdehyde (MDA) and hydrogen peroxide production were enhanced by NPs.	[233]
CeO_2_	Spinach *(Spinacia oleracea)*	Diameter of approximately 4 nm	Foliar exposure for 4 weeks (0.3 and 3 mg per plant).	Photosynthetic pigment content, plant biomass, lipid peroxidation, and plant biomass were not affected, while both doses caused downregulation of amino acids and reduction of Zn and Ca in leaves.	[234]
Y_2_O_3_	Maize *(Zea mays* L.*)*	NPs size (30 nm), hydrodynamic size (300.5 ± 14.1 nm) Zeta potential (5.27 ± 0.03 mV)	Seed germination in plastic tubes with concentrations of 10, 30, 50, 100, and 500 mg∙L^−1^, for 6 days	NPs had no effect on germination rates; peroxidase (POD) and catalase (CAT) were enhanced by NPs, polar metabolites showed a dose-dependent increase in NPs.	[235]

**Table 7:** Multiple findings toward metabolomics effects in plants due to carbon-based nanomaterials treatment.

NPs	Plant type	Nanoparticle characteristics	Experiential conditions	Effects	Ref
MWCNTs	Rose periwinkle (*Catharanthus roseus*)	Young’s modulus: 1200, tensile strength: 150, density 2.6 g∙cm^−3^, thermal conductivity: 3000 W∙m^−1^∙k^−1^, electron conductivity: 10^−5^-10^−7^ S∙m^−1^.	Seeds are grown in MS medium at 0, 50, 100, and 150 mg∙L^−1^	Increase in plant growth, biomass, root length, a slight increase in chlorophyll and carotenoids, increase in proteins, CAT, and POX enzymes.	[246]
MWCNTs	*Satureja khuzestanica*	Diameter of 15 nm and 50 μm.	Callus culture 0, 25, 50, 100, 250, and 500 μg∙mL^−1^	Enhanced flavonoids and phenols content in callus culture at 100 and 250 μg∙mL^−1^.	[247]
MWCNTs	*Thymus daenensis*	50 μm length, 233 m2∙g^−1^ surface area, 100 s∙cm^−1^ electrical conductivity, 3000 W∙m∙k^−1^.	MS media, 0, 125, 250, 500, 1000, and 2000 μg∙mL^−1^	Increased seedling biomass and height, highest total phenolic content, total flavonoid content, and antioxidant activity achieved with 250 μg∙mL^−1^.	[248]
GO	Gala apple *(Malus domestica)*	Particle diameter: 50–200 nm, thickness: 0.8–1.2 nm.	0, 0.1, 1, and 10 mg∙L^−1^ (40 days’ treatment)	Inhibition of lateral roots (0.1–10 mg∙L^−1^), GO increases CAT, POD, and SOD activities, 0.1 mg∙L^−1^ increases auxin efflux carrier and auxin influx genes transcription.	[249]
Single-bilayer GO	Faba bean (*Vicia faba* L.)	Size: 0.5–5 μm	0, 100, 200, 400, 800, and 1600 mg∙L^−1^	Decrease in growth, catalase, and ascorbate peroxidase activity, increase in electrolyte leakage.	[250]
Carbon nano-horns	*Arabidopsis thaliana*	Pipe diameter: 2–5, pipe length: 10–20 nm	0, 0.01, 0.05, 0.1, 0.3, 0.5, 1, 5, 10, 50, and 100 mg∙L^−1^	Single-wall carbon nano-horns altered sugar and amino acid content at 0.1 mg∙L^−1^ and increased secondary metabolites such as nicotinamide, purines, and flavones.	[251]
GO	Rice	Sheet thickness 1.12 nm, lateral length 0.5–2 μm	0.01–1.0 mg∙L^−1^	Upregulation of phenylalanine, secondary metabolism, inhibition of aquaporins.	[252]
